# Altered fecal microbiota composition in the Flinders sensitive line rat model of depression

**DOI:** 10.1007/s00213-018-5094-2

**Published:** 2018-11-23

**Authors:** Sandra Tillmann, Anders Abildgaard, Gudrun Winther, Gregers Wegener

**Affiliations:** 1 Translational Neuropsychiatry Unit, Department of Clinical Medicine, Aarhus University, Aarhus, Denmark; 2 Department of Clinical Biochemistry, Aarhus University Hospital, Aarhus, Denmark

**Keywords:** Depression, Gut-brain axis, Fecal microbiota transplantation, 16S rRNA amplicon sequencing

## Abstract

**Rationale:**

The gut microbiota is increasingly recognized as a potential mediator of psychiatric diseases. Depressed patients have been shown to have a different microbiota composition compared with healthy controls, and several lines of research now aim to restore this dysbiosis. To develop novel treatments, preclinical models may provide novel mechanistic insights.

**Objective and methods:**

We characterized the gut microbiota of male adult Flinders sensitive line (FSL) rats, an animal model of depression, and their controls, Flinders resistant line (FRL) rats using 16S rRNA amplicon sequencing. Moreover, we performed fecal microbiota transplantation (using saline or pooled FRL/FSL feces) to study if the potential strain-specific differences could be transferred from one strain to the other, and if these differences were reflected in their depressive-like behavior in the forced swim test.

**Results:**

FSL rats tended to have lower bacterial richness and altered relative abundances of several bacterial phyla, families, and species, including higher *Proteobacteria* and lower *Elusimicrobia* and *Saccharibacteria*. There was a clear separation between FRL and FSL rat strains, but no effect of treatment, i.e., the bacterial composition of FSL rats receiving FRL feces was still more similar to FSL and not FRL rats. Similarly, the transplantation did not reverse behavioral differences in the forced swim test, although FSL feces significantly increased immobility compared with saline.

**Conclusions:**

Our study showed that the gut microbiota composition of the depressive-like rats markedly differed from their controls, which may be of value for future microbiota-targeted work in this and similar animal models.

**Electronic supplementary material:**

The online version of this article (10.1007/s00213-018-5094-2) contains supplementary material, which is available to authorized users.

## Introduction

The gut microbiota is known to be involved in key physiological processes such as nutrient absorption, digestion, and metabolism. In addition to its homeostatic role, accumulating evidence suggests a widespread impact on human health and disease far beyond the gastrointestinal tract (Sekirov et al. 2010). These functions range from immune modulation to the development of distant tissues such as the bone, muscle, and brain, likely through bidirectional connections between the gut and extraintestinal organs (Fung et al. 2017). To exploit these communication systems for the benefit of human health, numerous studies in the past few decades have studied the effects of functional food supplements (e.g., prebiotics, probiotics, and synbiotics) and fecal microbiota transplantation (FMT).

Among many other observed health benefits, pre- and probiotics also positively affected mood and stress hormone concentrations in animals and healthy volunteers (Wallace and Milev, 2017, Wang et al. 2016, Dinan et al. 2013, Huang et al. 2016). In depressed patients, Akkasheh et al. (2015) found lower Beck Depression Inventory total scores after 8 weeks of probiotic supplementation, while Romijn et al. (2017) did not find evidence for antidepressant effects or changes in inflammatory markers. These studies differed in bacterial strain selection as well as disease severity and chronicity of the patient sample, which might explain conflicting results. Moreover, treatment response may have been affected by microbial group differences at baseline, as bacterial communities vary considerably according to the mode of delivery, host genetics, diet, health status, etc. (Rodriguez et al. 2015). In depressed patients, Jiang et al. (2015) found profound interindividual differences in alpha diversity according to treatment response and depression severity. Compared with healthy controls, depressed patients had decreased bacterial richness and diversity (Kelly et al. 2016). At the phylum level, they had increased relative abundances of *Actinobacteria* (Zheng et al. 2016, Jiang et al. 2015) and *Proteobacteria* (Jiang et al. 2015), and decreased *Firmicutes* (Jiang et al. 2015). Both increased (Jiang et al. 2015) and decreased (Zheng et al. 2016) levels of *Bacteroidetes* have been reported. Depressed patients also showed an increase in the order *Bacteroidales* and a decrease in the family *Lachnospiraceae* (Naseribafrouei et al. 2014). It is unknown whether these gut microbial changes reflect clinical symptoms and whether they are a cause or a result of the disease. Animal studies provided evidence to both, as stress caused gut microbial changes (O’Mahony et al. 2011, Bangsgaard Bendtsen et al. 2012, Abautret-Daly et al. 2018), Bailey et al. 2011) and gut microbiota transplantation from depressed patients induced depressive-like behavior in germ-free mice (Zheng et al. 2016) and microbiota-depleted rats (Kelly et al. 2016). These effects may be specific to microbiota-depleted models or human-derived feces, but they clearly demonstrate a causal association between gut microbiota and depressive-like behavior. To further explore this relationship, more data on conventional animals raised in non-sterile environments are needed.

Flinders sensitive line (FSL) rats show several behavioral and structural abnormalities compared with their control strain, Flinders resistant line (FRL) rats, and are used as an experimental animal model of depression (Overstreet and Wegener, 2013). Two recent studies in FSL rats found that probiotics protected against the pro-depressive-like effect of high-fat diet (Abildgaard et al. 2017) and induced changes in methyl donors and catecholamines (Tillmann et al. 2018). Given these data, further investigation of the gut-brain axis in these animals is of interest. To our knowledge, it is unknown if the gut microbiota composition of FSL rats differs from FRL controls, and how potential differences compare to depressed patients. However, this information may be useful in order to study causal mechanisms and to further investigate the gut-brain axis as a potential therapeutic target for depression. Our main objective was therefore to characterize the bacterial community of FSL vs. FRL rats using 16S rRNA amplicon sequencing to analyze whether their behavioral phenotype is related to potential differences in their gut microbiota composition. In addition, we performed FMT to investigate if potential microbial differences could be transplanted from one strain to the other and if these differences were reflected in their depressive-like behavior.

## Materials and methods

### Animals

Age-matched adult male Flinders sensitive line (FSL, *n* = 24) and Flinders resistant line (FRL, *n* = 24) rats were obtained from the breeding colony maintained at Aarhus University, Denmark. The day before the first transplantation, rats weighed 327.8 ± 40.7 g (mean ± SD) and were 10.6 ± 1.1 weeks old. Rats of the same strain and treatment were pair-housed in standard cages (Cage 1291H Eurostandard Type III H, 425 × 266 × 185 mm, Tecniplast, Italy) at 20 ± 2 °C and 60 ± 5% relative humidity on a reversed 12-h light/dark cycle (lights on at 2 p.m.). The reversed cycle was introduced in 3-h increments right before a 2-week acclimatization phase. After the acclimatization phase, rats were weighed once a week. All rats received standard chow diet (#1324 Altromin, Brogaarden, Lynge, Denmark) and tap water ad libitum and had access to a tunnel shelter, nesting material, and a wooden stick. Cages were changed once a week by the same experimenter performing the oral gavage and behavioral procedures. All experiments were approved by the Danish Animal Experiments Inspectorate prior to initiation of the experiments (approval number: 2012-15-2934-00254) and were conducted in accordance with the European Communities Council Directive.

### Experimental design

**Fig. 1:**
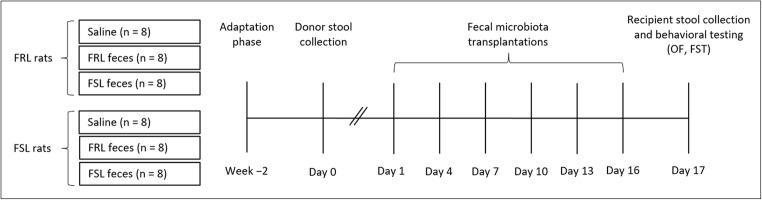
Experimental timeline. FRL and FSL rats were randomly distributed into three groups (saline, FRL feces, and FSL feces). After donor stool collection, rats received saline or pooled feces every third day for 16 days, amounting to six transplantations per animal. Recipient stool collection and behavioral testing took place on day 17. FRL, Flinders resistant line; FSL, Flinders sensitive line; FST, forced swim test; OF, open field

FRL rats were randomly assigned to one of three treatment groups: saline (vehicle control, *n* = 8), FRL feces (same-strain control, *n* = 8), or FSL feces (*n* = 8). Accordingly, FSL rats received saline (*n* = 8), FRL feces (*n* = 8), or FSL feces (*n* = 8). Saline controls were added to allow comparisons to a non-fecal group, whereas the same-strain comparisons (e.g., FSL feces transferred to FSL rats) enabled the distinction between feces derived from the two different strains (FSL/FRL). In order to reduce the number of animals in this study, the same rats were used as fecal donors and recipients. Donor stool was collected on day 0 between 2 and 2:30 p.m. Fecal suspensions or saline were administered every third day over a 16-day period (day 1, 4, 7, 10, 13, and 16) between 2 and 3 p.m., amounting to a total of six transplantations per rat. Each rat received 0.75 mL fecal solution or saline per transplantation using oral gavage. Recipient stool collection and behavioral testing took place on day 17. A graphical representation of the experimental design is provided in Fig. 1.

### Fecal material preparation

The day before the first transplantation, fresh fecal pellets were taken from the rectal ampulla of the rats and kept on wet ice immediately following collection. Feces from individual FRL rats were pooled to increase diversity and sample volume (the pool is hereafter referred to as “FRL feces”). Individual FSL fecal pellets were also pooled accordingly (“FSL feces”). Fecal material was prepared according to the European Consensus Conference for FMT in clinical practice (Cammarota et al. 2017), which was adapted for our study. The guidelines recommend 30 g of donor feces per person and transplantation to be diluted with sterile saline with 3–5 times larger volume of solvent (e.g., 30 g of feces diluted in 150 mL of saline). Assuming an average human weight of 70 kg, this is equivalent to 0.43-g feces per kg bodyweight. Accordingly, a 350-g rat should receive 0.15-g feces diluted in 0.75 mL saline. Our fecal collection yielded approximately 25 g of fecal material per pool, which was homogenized in 125 mL saline using a standard commercial blender. Before freezing at − 80 °C, 85% glycerol (Sigma-Aldrich, Steinheim, Germany) was added to a final concentration of 10% as per guideline recommendations. To avoid multiple freeze-thaw cycles, six aliquots per FRL/FSL pool for the six planned transplantations were prepared. On transplantation days, the designated aliquots were thawed in a warm (37 °C) water bath over 2 h immediately prior to transplantation. Rats were administered 0.75 mL fecal suspension per transplantation (consisting of 0.15 g pooled feces) using oral gavage.

### DNA extraction

Fecal pellets were collected on day 17 prior to behavioral testing and immediately frozen at − 80 °C. DNA was extracted using QIAamp PowerFecal DNA Kit (including a bead-beating step; #12830-50; Qiagen, Germany) according to the manufacturer’s instructions and sent to DNASense (Aalborg, Denmark) for 16S rRNA amplicon sequencing.

### Library preparation

Bacterial V4 16S sequencing libraries were prepared using a custom protocol based on an Illumina protocol (#15044223 Rev. B, Illumina, San Diego, CA, USA). Up to 10 ng of extracted DNA was used as a template for PCR amplification of the 16S gene fragments. Each PCR reaction (25 μL) contained dNTPs (100 μM of each), MgSO4 (1.5 mM), Platinum® Taq DNA polymerase HF (1 U), 1× Platinum® High Fidelity buffer (Thermo Fisher Scientific, Oakwood, OH, USA), and tailed primer mix (400 nM of each forward and reverse). PCR was run with the following program: initial denaturation at 95 °C for 2 min, 35 cycles of amplification (95 °C for 20 s, 50 °C for 30 s, 72 °C for 60 s), and a final elongation at 72 °C for 5 min. Duplicate PCR reactions were performed for each sample and the duplicates were pooled after PCR. The forward and reverse tailed primers were designed according to the Illumina protocol (#15044223 Rev. B, 2015) and contained primers targeting the bacteria and archaea 16S gene V4 region (Caporaso et al. 2011): 5′-GTGCCAGCMGCCGCGGTAA (515F) and 5′-GGACTACHVGGGTWTCTAAT (806R). The primer tails enable attachment of Illumina Nextera adaptors for sequencing in a subsequent PCR. The resulting amplicon libraries were purified using the standard protocol for Agencourt Ampure XP Bead (Beckman Coulter, Brea, CA, USA) with a modified bead-to-sample ratio of 4:5. The DNA was eluted in 33 μL of nuclease-free water (Qiagen, Hilden, Germany). DNA concentration was measured using Qubit™ HS DNA Assay kit (Thermo Fisher Scientific, USA). Sequencing libraries were prepared from the purified amplicon libraries using a second PCR. Each PCR reaction (25 μL) contained 1× PCRBIO HiFi buffer (PCR Biosystems, London, UK), PCRBIO HiFi Polymerase (1 U) (PCR Biosystems, UK), adapter mix (400 nM of each forward and reverse), and up to 10 ng of amplicon library template. PCR was run with the following program: initial denaturation at 95 °C for 2 min, 8 cycles of amplification (95 °C for 20 s, 55 °C for 30 s, 72 °C for 60 s) and a final elongation at 72 °C for 5 min. The resulting sequencing libraries were purified using the standard protocol for Agencourt Ampure XP Bead (Beckman Coulter, USA) with a modified bead-to-sample ratio of 4:5. The DNA was eluted in 33 μL of nuclease-free water (Qiagen, Germany). DNA concentration was measured using Qubit™ HS DNA Assay kit (Thermo Fisher Scientific, USA). Gel electrophoresis using Tapestation 2200 and D1000 screentapes (Agilent, Lexington, MA, USA) was used to check the product size and purity of a subset of sequencing libraries. Double-indexing was performed on the libraries.

### DNA sequencing

The purified sequencing libraries were pooled in equimolar concentrations and diluted to 2 nM. The samples were sequenced (2 × 301 bp) on a MiSeq (Illumina, USA) using a MiSeq Reagent Kit v3 (Illumina, USA) following the standard guidelines for preparing and loading samples on the MiSeq. To overcome low complexity issue often observed with amplicon samples, 20% Phix control library was spiked in.

### Bioinformatic processing

Demultiplexing was done by the MiSeq software. Forward and reverse reads were trimmed for quality using Trimmomatic v. 0.32 (Bolger et al. 2014) with the settings SLIDINGWINDOW:5:3 and MINLEN:250. The trimmed forward and reverse reads were merged using FLASH v. 1.2.7 (Magoc and Salzberg, 2011) with the settings -m 10 -M 200. The merged reads were dereplicated and formatted for use in the UPARSE workflow (Edgar, 2013). Chimeras were removed as a de novo step in the UPARSE pipeline by default. The dereplicated reads were clustered using the usearch v. 7.0.1090 -cluster_otus command with default settings. Operational taxonomic unit (OTU) abundances were estimated using the usearch v. 7.0.1090 -usearch_global command with -id 0.97. Taxonomy was assigned using the RDP classifier (Wang et al. 2007) as implemented in the parallel_assign_taxonomy_rdp.py script in QIIME (Caporaso et al. 2011), using the MiDAS database v.1.23 (McIlroy et al. 2015). The results were analyzed in R through the Rstudio IDE using the ampvis package v.2.2.6 (Albertsen et al. 2015). Rarefaction curves were checked and showed a very low diversity community with the expected flattening curves (Supplementary Fig. 1).

### Behavioral analysis

All behavioral tests were performed in specially equipped rooms within the animal facility between 08:00 a.m. and 12:30 p.m. in the active phase of the animal under dim red light. The order of animals was determined randomly. To minimize stress, animals were habituated to the behavioral rooms 1 h before testing commenced. All arenas/tanks were thoroughly cleaned between each trial. Tests were scored by an observer blinded to strain and treatment groups.

#### Open field

To measure locomotion, rats were placed in an open-field arena (100 × 100 × 80 cm) immediately prior to the forced swim test as described previously (Tillmann et al. 2017). Briefly, animals were placed in the center of the square and allowed to move freely for 5 min, which was recorded by a camera mounted to the ceiling. The total distance traveled (cm) was obtained with specialized software tracking the midpoint of the rats’ body contour (Ethovision XT, Noldus Information Technology, Wageningen, Netherlands).

#### Forced swim test

To measure depressive-like behavior, the forced swim test (28) was employed as described previously (Tillmann et al. 2017). Briefly, rats were placed into a perspex cylinder (height, 60 cm; diameter, 24 cm) filled with 24 (± 1)°C heated tap water for 5 min. A camera was positioned in front of the water tanks to record their behavior. Three distinct behaviors were scored, including struggling (vertical movements of the forepaws), swimming (horizontal movements), and immobility (floating posture with minimal movements), whereby immobility was interpreted as depressive-like behavior (Slattery and Cryan, 2012).

### Data analysis

Bodyweight and behavioral data were analyzed by a two-way ANOVA (strain × treatment). In case of a statistically significant treatment effect or interaction, these were followed up by the Bonferroni-corrected comparisons. Assumptions of normality and homogeneity of variances were tested by the Shapiro-Wilk test and Levene’s test, respectively. Data in the figures are presented as means ± standard error of the mean (SEM). Alpha was set at 0.05, while a *p* value between 0.05 and 0.1 was interpreted as a trend. Statistical analyses were carried out using IBM SPSS 22.0 (IBM Corp., Armonk, NY, USA). Graphs were generated using SPSS and GraphPad Prism 5.0 (GraphPad Software Inc., San Diego, CA, USA).

Relative abundances (log-transformed) at the phylum and family level were analyzed by mixed model regression: rat identity comprised the random effect (intercept), whereas fixed effects included rat strain, treatment, phylum/family, and their interactions. To investigate any associations with depressive-like behavior, the animals were divided into quartiles based on immobility in the forced swim test, and the forced swim test quartile was included in the model. Families/phyla not observed in > 50% of animals were excluded. Restricted maximum likelihood (REML) estimation was used, and residuals were considered independent by phylum/family. Contrasts of marginal linear predictions were used to test for differences between groups. The results are expressed as relative abundances with 95% CI, and analyses were performed with and without the Bonferroni correction as stated. Mixed model regression was performed with Stata 14 (StataCorp LT, TX, USA). At the OTU level, differential abundance was analyzed by DESeq2 (differential expression analysis based on the negative binomial distribution), and *p* values were adjusted by false discovery rate (FDR) according to Benjamini and Hochberg (Benjamini and Hochberg, 1995). In addition, the overall microbiota composition was analyzed by a principal component analysis (PCA) and a partial least-squares discriminant analysis (PLS-DA) with pareto scaling of the OTUs and a missing value tolerance of 50% with Simca 14 (MKS Umetrics AB, Sweden). The relationship between the first component (PC1) of the PLS-DA analysis and immobility behavior of the two strains was assessed by Spearman’s rank correlation.

## Results

### Body weight and water/food intake

FSL rats weighed significantly less than FRL rats at both baseline (day 0; means ± SD, 301.6 ± 28.2 g vs. 354.0 ± 34.1 g; *F*(1,42) = 32.97, *p* < 0.001) and endpoint (day 17; 340.0 ± 27.1 g vs. 397.7 ± 34.6 g; *F*(1,42) = 39.51, *p* < 0.001), which is characteristic for their depressive-like phenotype (Overstreet, 1993). Treatment groups did not differ in body weight at baseline or endpoint (*p*’s > 0.355), and there was no interaction between strain and treatment (*p*’s > 0.676). There were no significant differences in water or food intake (grams of food per 100 g body weight) between strains or treatment groups (*p*’s > 0.181).

### Gut microbiota analysis

DNA extraction was successful and yielded similar DNA concentrations for most samples (57.8 ± 3.5 ng/μL). Sample preparation for bacterial sequencing (V4) was also successful for all samples and yielded between 11,954 and 172,082 reads after quality control and bioinformatic processing. One sample (strain: FRL, treatment: saline) was unsuccessful and excluded from the microbiota results. All OTUs are provided in Supplementary Table 1.

#### Reduced gut microbiota richness in FSL rats

**Fig. 2:**
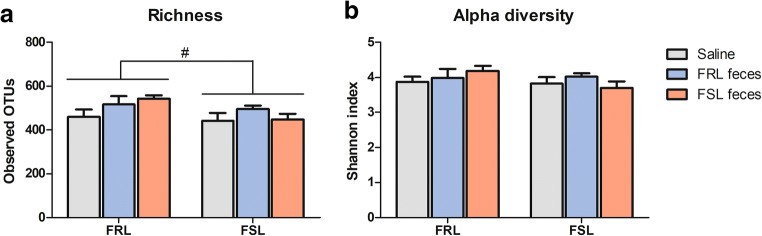
Gut microbiota richness and diversity following fecal transplantation. **a** FSL rats tended to have lower gut microbiota richness than FRL rats (*p* = 0.066), but there were no treatment or interaction effects**. b** Alpha diversity did not differ between strains or treatment groups. Values are expressed as means ± SEM and were analyzed using a two-way ANOVA; *n* = 8/group, except for FRL saline (*n* = 7). FRL, Flinders resistant line; FSL, Flinders sensitive line; #*p* = 0.066

Compared with FRL rats, FSLs tended to have lower gut microbiota richness as calculated by the number of observed OTUs based on 10.000 reads per sample (507.8 ± 87.2 vs. 461.7 ± 76.7; *F*(1,41) = 3.56, *p* = 0.066, Fig. 2a). There were no treatment or interaction effects (*p*’s > 0.153). The Shannon index, a measure of alpha diversity, did not significantly differ between strains or treatment groups (*p*’s > 0.276, Fig. 2b).

#### Multivariate inference revealed a clear separation between FSL and FRL rats

**Fig. 3:**
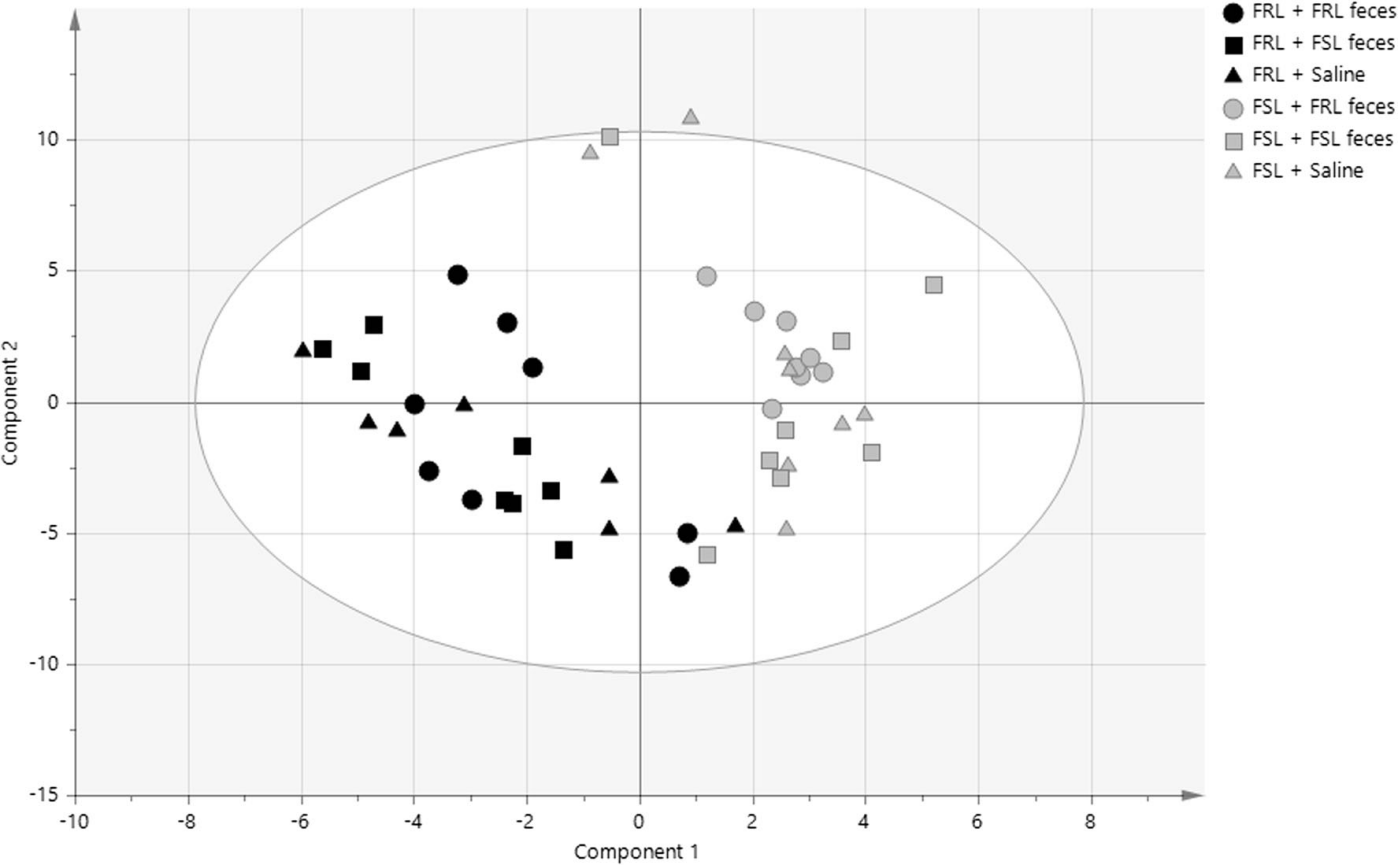
PLS-DA analysis of fecal microbiota. A clear separation between FRL and FSL rat strains was evident (*R*^2^(*Y*) = 0.8; *Q*^2^ = 0.7), but the model was not able to predict treatment

To assess whether the microbiota could predict group belonging, we performed a PLS-DA analysis. A model that included all six groups as identifiers yielded a poor predictive power (*R*^2^(*Y*) = 0.1; *Q*^2^ = 0.05). However, when only rat strain was used as a class identifier, an improved model was constructed (*R*^2^(*Y*) = 0.8; *Q*^2^ = 0.7), and a permutation test corroborated the ability of the model to predict rat strain on the basis of the gut microbiota. Indeed, a clear separation between FRL and FSL rats was seen along the first component (*R*^2^ = 0.136) (Fig. 3). A separation between FRL and FSL rats was also observed on a PCA plot along the second component (*R*^2^ = 0.087) (Supplementary Fig. 2). In conclusion, the overall structure of the gut microbiota was found to differ between FRL and FSL rats, but was not altered by treatment.

#### Phylum-level differences between strains and treatment groups

**Fig. 4:**
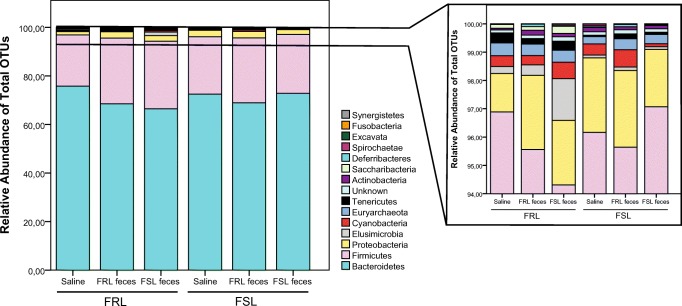
Summed relative abundance of total OTUs at phylum level following fecal transplantation. The right graph represents a zoomed-in view of phyla between 94 and 100%. Phyla with an abundance below 0.001% were excluded from analyses and graphs; *n* = 8/group, except for FRL saline (*n* = 7). FRL, Flinders resistant line; FSL, Flinders sensitive line

Microbiota composition at the phylum level is depicted in Fig. 4. *Bacteroidetes* represented the highest abundance in all samples (total mean, 69.9% (95% CI, 67.2–72.8)), followed by *Firmicutes* (23.4% (20.8–26.4)) and *Proteobacteria* (2.05% (1.79–2.34)). Compared with FRL rats, FSLs had a lower abundance of *Elusimicrobia* (0.26% (0.15–0.44) vs. 0.08% (0.05–0.13); *χ*^2^ = 9.03; *p* = 0.003) and *Saccharibacteria* (0.10% (0.05–0.21) vs. 0.00% (0.00–0.01); *χ*^2^ = 35.3; *p* < 0.0001), and these differences remained statistically significant after the Bonferroni correction (*p*’s < 0.032). Conversely, the abundance of *Proteobacteria* was higher in FSL rats than in FRL rats (2.37% (1.94–2.89) vs. 1.72% (1.40–2.10); *χ*^2^ = 4.30; *p* = 0.04), although this finding did not pass the Bonferroni correction (*p* = 0.7).

Two interactions between strain and treatment were observed before the Bonferroni correction was applied. In the *Deferribacteres* phylum (interaction, *χ*^2^ = 6.03; *p* = 0.049), treatment did not affect FSL rats (*χ*^2^ = 5.74; *p* = 0.28 (Bonferroni)), whereas a higher abundance was seen in FRL rats receiving FRL feces than in FRL rats receiving saline (0.01% (0.01–0.03) vs. 0.06% (0.03–0.12); *z* = 3.09; *p* = 0.006 (Bonferroni)). The second interaction was found in the *Cyanobacteria* phylum (interaction *χ*^2^ = 9.34; *p* = 0.009), but no follow-up tests reached statistical significance, although FSL feces treatment tended to cause lower *Cyanobacteria* abundance in FRL rats than in FSL rats (FRL receiving FSL feces, 0.40% (0.15–1.08); FSL receiving FSL feces, 0.07% (0.02–0.20); *χ*^2^ = 5.64; *p* = 0.09 (Bonferroni)). Treatment alone was not found to affect the microbiota composition at the phylum level. There were no main or interaction effects in *Firmicutes:Bacteroidetes* or *Actinobacteria:Proteobacteria* ratios (*p*’s > 0.330).

#### Family-level differences between strains and treatment groups

**Fig. 5:**
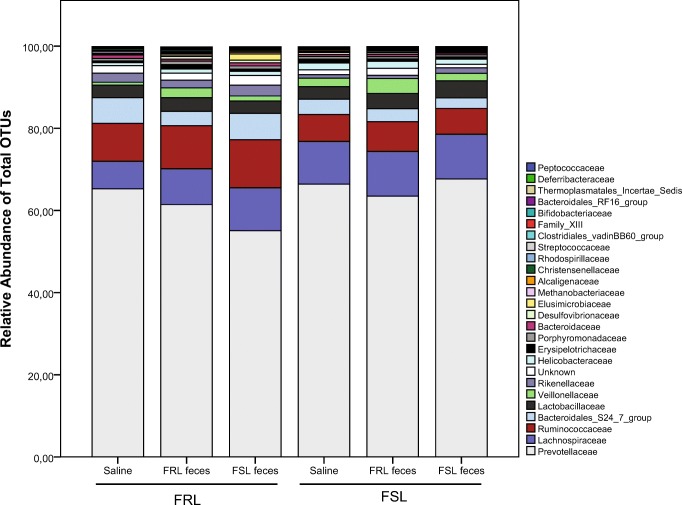
Summed relative abundance of the 25 most abundant OTUs at family level following fecal transplantation; *n* = 8/group, except for FRL saline (*n* = 7). FRL, Flinders resistant line; FSL, Flinders sensitive line

*Prevotellaceae* represented the highest percentage in all samples (total mean, 61.8% (51.0–74.8)), followed by *Lachnospiraceae* (8.73% (7.60–10.0)) and *Ruminococcaceae* (7.46% (6.46–8.61)). The 25 most abundant OTUs (representing 99.85% of the sequence reads) are reported in Fig. 5.

FSLs had a significantly higher abundance of *Bifidobacteriaceae* (0.05% (0.03–0.10) vs. 0.02% (0.01–0.03); *χ*^2^ = 11.5; *p* = 0.0007), but a lower abundance of *Rikenellaceae* (0.81% (0.62–1.06) vs. 1.87% (1.42–2.45); *χ*^2^ = 18.6; *p* < 0.0001), *Elusimicrobiaceae* (0.08% (0.05–0.13) vs. 0.26% (0.16–0.44); *χ*^2^ = 10.8; *p* = 0.001), *Christensenellaceae* (0.09% (0.07–0.13) vs. 0.28% (0.20–0.40); *χ*^2^ = 17.5; *p* < 0.0001), and *Peptostreptococcaceae* (0.01% (0.00–0.01) vs. 0.03% (0.02–0.05); *χ*^2^ = 19.3; *p* < 0.000) than FRL rats. All these alterations remained statistically significant after the Bonferroni correction (*p*’s < 0.037).

Without the Bonferroni correction, FSLs also had a lower abundance of *Bacteroidales S24-7 group* (2.74% (2.15–3.50) vs. 4.44% (3.46–5.69); *χ*^2^ = 4.62; *p* = 0.03), *Peptococcaceae* (0.03% (0.02–0.04) vs. 0.04% (0.03–0.06); *χ*^2^ = 4.55; *p* = 0.03), and *Eubacteriaceae* (0.001% (0.001–0.002) vs. 0.002% (0.002–0.003); *χ*^2^ = 3.91; *p* = 0.048), but a higher abundance of *Lactobacillaceae* (3.38% (2.66–4.29) vs. 2.79% (2.19–3.56); *χ*^2^ = 3.99; *p* = 0.046), *Lachnospiraceae* (9.58% (7.69–11.94) vs. 7.92% (6.32–9.91); *χ*^2^ = 5.85; *p* = 0.02), *Veillonellaceae* (2.06% (1.39–3.04) vs. 0.94% (0.63–1.40); *χ*^2^ = 8.98; *p* = 0.003), and *Helicobacteraceae* (1.37% (1.01–1.85) vs. 0.76% (0.56–1.03); *χ*^2^ = 7.00; *p* = 0.008).

Treatment affected several microbial families. A marked treatment effect was seen for *Rhodospirillaceae* (*χ*^2^ = 20.4; *p* < 0.0001). Specifically, FRL feces-treated rats had a higher abundance than saline-treated rats (0.15% (0.08–0.30) vs. 0.02% (0.01–0.05); *z* = 4.51; *p* < 0.001 (Bonferroni)), and the same was the case for treatment with FSL feces (0.07% (0.03–0.13); *z* = 2.53; *p* = 0.03 (Bonferroni)). However, FSL and FRL feces groups did not differ (*z* = 1.83; *p* = 0.2 (Bonferroni)). In addition, three other families were affected by treatment, although these changes did not remain statistically significant after the Bonferroni correction: For *Bifidobacteriaceae* (*χ*^2^ = 7.56; *p* = 0.02), FRL feces led to a higher abundance than saline (0.05% (0.02–0.10) vs. 0.02% (0.01–0.04); *z* = 2.52; *p* = 0.04 (Bonferroni)), whereas the effect of FSL feces narrowly missed significance in comparison with saline (0.04% (0.02–0.08); *z* = 2.36; *p* = 0.06 (Bonferroni)). FSL and FRL feces groups did not differ (*z* = 0.00; *p* = 1.0). For *Deferribacteraceae* (*χ*^2^ = 10.5; *p* = 0.005), treatment with FRL feces caused higher levels than treatment with FSL feces or saline (saline, 0.02% (0.01–0.04); FRL feces, 0.06% (0.04–0.10); FSL feces, 0.03% (0.02–0.05); *p*’s < 0.02 (Bonferroni)). Similarly, FRL feces caused higher abundance of *Peptostreptococcaceae* (*χ*^2^ = 8.23; *p* = 0.02) compared with FSL feces and saline (saline, 0.01% (0.00–0.02); FRL feces, 0.03% (0.01–0.05); FSL feces, 0.01% (0.00–0.02); *p*’s < 0.05 (Bonferroni)).

A single treatment × strain interaction was found for OTUs belonging to unclassified families (*χ*^2^ = 9.67; *p* = 0.008), but only before the Bonferroni correction was applied. Specifically, FSL feces led to different abundancies in the two strains (FRLs receiving FSL feces, 2.20% (1.54–3.15) vs. FSLs receiving FSL feces, 0.83% (0.56–1.23); *χ*^2^ = 12.8; *p* = 0.002 (Bonferroni)).

#### OTU-level differences between FRL and FSL

Since the PLS-DA analysis revealed a clear effect of strain on the gut microbiota composition, we also compared FRL rats with FSL rats on the lowest taxonomic level, the OTU level. In total, 475 OTUs were detected, and 167 (35%) of these were found to differ between the rat strains after statistical correction (Supplementary Table 2). Most of the significantly different OTUs were found at a higher level in FRL rats (109), whereas only 58 were found at a higher level in FSL rats.

Among the significantly different OTUs between FSL and FRL rats, FSLs had lower relative abundances of all 15 OTUs from the *Bacteroidales S24-7* family, all four OTUs from the *Candidatus Saccharimonas* genus, both OTUs from the *Alistipes* genus, and both OTUs from the *Roseburia* genus.

FSL rats had higher abundances of all six OTUs from the *Blautia* genus and all four OTUs from the *Subdoligranulum* genus. Of the three significantly different OTUs from the *Lactobacillus* genus, two were found in lower relative abundances in FSL rats and one in higher abundance. There was no significant difference in *Bifidobacteria*.

### Behavioral results

**Fig. 6:**
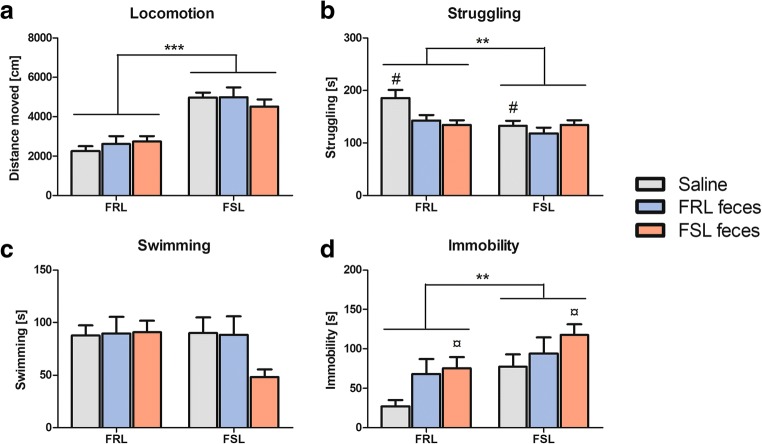
Behavioral results following fecal transplantation. **a** FSL rats traveled a significantly greater distance than FRL rats in a 5-min open-field session (*p* < 0.001). **b** FSL rats struggled significantly less than FRL rats in a 5-min forced swim test session (*p* = 0.007). FRL feces administration decreased struggling behavior compared with saline (*p* = 0.041). **c** There were no significant main or interaction effects in swimming behavior. **d** FSLs were significantly more immobile than FRLs (*p* = 0.004). FSL feces administration increased immobility compared with saline (*p* = 0.023). Values are expressed as means ± SEM and were analyzed by a two-way ANOVA followed by post hoc comparisons; *n* = 8/group. FRL, Flinders resistant line; FSL, Flinders sensitive line; **p < 0.01, ***p < 0.001; #, significant treatment effect compared with FRL feces; ¤, significant treatment effect compared with saline

In the open field, FSL rats moved a significantly greater total distance than FRL rats (4826 ± 1075 cm vs. 2543 ± 868 cm; *F*(1,42) = 63.06, *p* < 0.001, Fig. 6a). There were no treatment or interaction effects (*p*’s > 0.404).

#### Fecal transplantation altered behavior in the forced swim test

In the forced swim test, FSL rats struggled significantly less than FRL rats (128 ± 28 s vs. 154 ± 40 s; *F*(1,42) = 8.03, *p* = 0.007, Fig. 6b) and were more immobile (96.3 ± 48.6 s vs. 56.7 ± 44.7 s; *F*(1,42) = 9.51, *p* = 0.004, Fig. 6d). The strains did not differ in their total swimming duration (*p* = 0.213, Fig. 6c). There were significant treatment effects in struggling (*F*(2,42) = 3.88, *p* = 0.028) and immobility (*F*(2,42) = 4.05, *p* = 0.025). Bonferroni multiple comparisons of the treatment groups revealed that rats receiving FRL feces struggled less than saline-treated ones (*p* = 0.041), while there was no difference between FSL feces and saline or FSL and FRL feces (*p*’s > 0.098). There was a trend towards a strain × treatment interaction in struggling (*F*(2,42) = 2.84, *p* = 0.070). FSL feces administration significantly increased immobility compared with saline (*p* = 0.023), whereas FRL feces did not differ from saline (*p* = 0.224). There was no difference in immobility between FSL and FRL feces (*p* = 1.00).

### Associations between gut microbiota composition and behavior

To explore whether the behavioral difference between FSLs and FRLs may be linked to their gut microbial composition, we analyzed the relationship between immobility and the first component (PC1) of the PLS-DA analysis, since this component represented the composite microbial pattern that ultimately separates the two strains. However, no correlation was evident between PC1 and FRL immobility scores (*r* = 0.034, *n* = 23, *p* = 0.877) or FSL immobility scores (*r* = 0.103, *n* = 24, *p* = 0.631).

In addition, we examined correlations between immobility and the 20 individual OTUs that were best at separating FSL and FRL rats, but found no significant effect (data not shown).

We also divided the animals into quartiles based on their immobility duration and analyzed their relationship with phylum and family abundancies in the mixed regression model. At the phylum level, no significant associations were seen between quartile of immobility and microbial abundancies. At the family level, however, *Alcaligenaceae* abundance decreased with increasing immobility levels (Q1, 0.39% (0.28–0.55); Q2, 0.28% (0.23–0.34); Q3, 0.20% (0.16–0.24); Q4, 0.14% (0.10–0.20); *z* = −3.41; *p* = 0.02 (Bonferroni)).

## Discussion

Our findings demonstrate that the gut microbiota composition of the depressive-like FSL rats significantly differed from control FRL rats. FSL rats had not only a tendency to a lower number of OTUs but also altered relative abundances of several phylum-, family-, and species-level bacteria. Fecal transplantation with pooled FSL or FRL feces did not reverse these bacterial differences. On a behavioral level, FSL feces significantly increased immobility in the forced swim test compared with saline but not compared with FRL feces.

### Microbial richness and diversity

Lower bacterial richness as observed in FSL rats was also found in depressed patients compared with healthy controls (Kelly et al. 2016). However, it must be noted that the richness difference in our study was largely driven by elevated levels in FRL rats receiving pooled feces rather than saline, suggesting that treatment-naïve FSL and FRL rats may not differ in bacterial richness. A lack of difference in species richness between depressed patients and controls was also found in several human studies (Naseribafrouei et al. 2014, Jiang et al. 2015). We found no evidence for altered alpha diversity levels between FSL and FRL rats, and human data are inconclusive in that regard, with studies reporting both increased (Jiang et al. 2015), decreased (Kelly et al. 2016), or similar (Naseribafrouei et al. 2014) diversity in depressed vs. non-depressed participants. Conflicting studies may be due to heterogeneous patient or control groups, analytical methods, confounding medication, diet, or a combination thereof. Data on microbial richness and diversity in animal models of depression are sparse, but a recent study in mice reported reduced richness and diversity following social defeat (Bharwani et al. 2016). Galley et al. (2014) further noted that restraint stress in mice caused reduced alpha diversity in colonic mucosa- but not luminal-associated communities, whereas richness was not reduced in either microbial niche. Park et al. (2013) demonstrated that experimentally induced depression in mice through olfactory bulbectomy changed the composition of the microbiota, but the authors did not assess richness or diversity.

### Gut microbiota composition in FSL vs. FRL rats

We were able to differentiate FSL from FRL rats on the basis of their gut microbiota using PLS-DA analysis (*R*^2^(*Y*) = 0.8; *Q*^2^ = 0.7). When fecal transplantation treatment groups were included as identifiers, the predictive value decreased substantially (*R*^2^(*Y*) = 0.1; *Q*^2^ = 0.05), indicating that the model was able to predict rat strain but not treatment. Similarly, we found several strain differences between FSL and FRL rats on phylum, family, and species levels, but only limited effects of treatment. On the phylum level, FSL rats had significantly lower relative abundances of *Elusimicrobia* and *Saccharibacteria* than FRLs. Before the Bonferroni correction, FSLs had higher abundances of *Proteobacteria*. Interestingly, Jiang et al. (2015) corroborated this finding in depressed patients, suggesting that the overrepresentation of *Proteobacteria* may be associated with depression. A recent review further noted that these gram-negative bacteria are often increased in intestinal and extraintestinal diseases with an inflammatory phenotype, and that they may serve as a potential diagnostic marker of dysbiosis and risk of disease (Rizzatti et al. 2017). Given the close connection between depression and inflammation (Miller and Raison, 2016), future causal studies of the relationship between *Proteobacteria* and depression are warranted.

On the family level, FSLs had a lower relative abundance of *Rikenellaceae*, *Elusimicrobiaceae*, *Christensenellaceae*, and *Peptostreptococcaceae*. Before the Bonferroni correction, they also had lower abundances of *Bacteroidales S24-7 group*, *Peptococcaceae*, and *Eubacteriaceae*. Interestingly, *Rikenellaceae* has previously been associated with fear (Christian et al. 2015) and depression (Jiang et al. 2015) in humans, and its member *Alistipes* was found to be increased in stress-exposed mice (Bangsgaard Bendtsen et al. 2012), indicating that this family may be involved with psychiatric symptomatology. *Bacteroidales S24-7 group* was found to be reduced in mice following restrained stress (Wong et al. 2016). FSLs had higher relative abundances of *Bifidobacteriaceae* and, before Bonferroni correction, *Lactobacillaceae*, *Lachnospiraceae*, *Veillonellaceae*, and *Helicobacteraceae*. In mice, *Lachnospiraceae* was found to be increased following restraint stress (Wong et al. 2016), whereas human studies reported lower relative abundances in depressed patients compared with healthy controls (Jiang et al. 2015, Naseribafrouei et al. 2014).

At the OTU level, FSL rats had lower relative abundances of the genera *Candidatus Saccharimonas*, *Alistipes*, and *Roseburia*. Previous work showed that mice treated with 5-fluorouracil, a model of intestinal mucositis, had decreased abundances of *Candidatus Saccharimonas* (Li et al. 2017), which may be indicative of intestinal dysbiosis in FSL rats. The *Alistipes* genus has previously been found to be overrepresented in depressed patients (Naseribafrouei et al. 2014, Jiang et al. 2015), which is in contrast to our findings in the depressed rat model. Several species of the *Roseburia* genus have been suggested as a marker of health due to their butyrate-producing properties (Tamanai-Shacoori et al. 2017). Butyrate is one of the three major short-chain fatty acids and is generally associated with positive effects on health (Canani et al. 2011). An underrepresentation of *Roseburia* in FSL rats may thus be indicative of decreased butyrate production, and this should be explored by future studies. FSLs had higher relative abundances of *Blautia* and *Subdoligranulum* genera. The *Blautia* genus was found to be overrepresented in depressed patients compared to controls (Jiang et al. 2015), which is in line with our findings. Two of the three significantly different OTUs from the *Lactobacillus* genus were found in lower abundances in FSL rats, whereas one was found in higher abundances compared to FRL rats. *Lactobacilli* are usually associated with health benefits and a depletion may be correlated to a depressive-like phenotype (Marin et al. 2017, Aizawa et al. 2016). *Bifidobacteria* also generally exert health-promoting effects (O’Callaghan and van Sinderen, 2016), but we did not find any significant differences in FSL vs. FRL rats.

### Effects of fecal transplantation on gut microbiota and behavior

Fecal transplantation from FSL to FRL rats and vice versa did not appear to shift the strain-characteristic phyla- and family-level differences; for instance, the increased relative abundance of *Proteobacteria* found in FSL rats did not decrease upon transplantation with FRL feces. Interestingly, *Elusimicrobia*/*Elusimicrobiaceae* seemed to be increased in FRL rats receiving FSL feces, but not in FSL rats, although both received the same pooled fecal suspension, and although these bacteria were only present in FSL rats in low abundances. Similar observations can be made for *Saccharibacteria*. FSL feces also lowered the abundance of *Cyanobacteria* in FRL but not FSL rats. FRL rats may therefore be more receptive to the introduction of these bacteria, but the functional consequences remain elusive. Administration of FRL feces caused a higher relative abundance of several bacterial families, such as *Rhodospirillaceae*, *Bifidobacteriaceae*, *Deferribacteraceae*, and *Peptostreptococcaceae*. Increased abundance of bacteria upon the introduction of feces independent of the origin would perhaps not be surprising, but the distinct strain-dependent increase in FRL feces compared with FSL feces warrants further investigation.

Behavioral analyses confirmed the depressive-like phenotype of FSL rats in the forced swim test, in that FSLs were more immobile and struggled less than FRL rats (Overstreet and Wegener, 2013). Transplantation with FSL feces increased immobility, supporting a transmissible behavioral phenotype. However, the effect was only significant compared with saline and not compared with FRL feces, limiting the overall generalizability of this finding. Contrary to our initial hypothesis, transplantation with FRL feces did not decrease immobility in FSL rats. According to our microbiota analyses, the identified characteristic bacteria for the strains were not transmissed, which might explain the limited behavioral effect. Zheng et al. (2016) found alterations in the forced swim test following FMT from depressed patients to germ-free mice. In contrast, although Kelly et al. (2016) we are able to shift the bacterial composition in microbiota-depleted rats following FMT from depressed patients, they did not observe concurrent behavioral changes in the forced swim test, implying that some neurobiological correlates of depression may not be transmissible through FMT. Other potentially relevant behavioral tests (i.e., anhedonia, cognition, and anxiety) were not conducted in the present study because FSL and FRL rats do not usually differ in these domains (Overstreet and Wegener, 2013, Tillmann et al. 2018), and a behavioral transmission from one strain to another would therefore not be of immediate interest. Fecal treatment in general seemed to decrease struggling in FRL rats compared with saline, again suggesting that FRL rats may be more susceptive to the introduction of certain bacteria and hence may possess a more malleable microbiota than FSLs. However, since feces contain significant amounts of non-bacterial matter (e.g., colonocytes, archaea, viruses, fungi, protists, and metabolites (Bojanova and Bordenstein, 2016)), the effects of the bacterial transplant alone cannot be separated, and conclusions should be drawn with caution.

To further investigate if the behavioral difference between FSL and FRL rats may be linked to their gut microbiota, we correlated immobility to the first component of the PLS-DA analysis, the component that ultimately separated the two strains. We also correlated immobility to the 20 individual OTUs that were best at separating the strains, but found no effects. Interestingly, *Alcaligenaceae* abundance decreased with increasing immobility levels, i.e., animals that were more immobile had a lower relative abundance of the bacteria. *Alcaligenaceae* was previously found to be increased in sedentary rats selectively bred for low-capacity running (Panasevich et al. 2016) as well as rats on a high-fat diet (Khan et al. 2018), suggesting a metabolic component which may also be relevant to consider in case of the forced swim test given the difference in energy effort between immobile and struggling rats. This family has also been positively associated with cognitive decline in cirrhosis patients (Bajaj et al. 2012), i.e., lower abundance reflected better memory.

### Strengths and limitations

While our study has significant strengths such as the use of non-sterile animals, an additional non-fecal control group, and long-term FMT treatment, several limitations should be mentioned. Our FMT protocol was based on the human consensus guidelines (Cammarota et al. 2017) and on previous attempts in rodents (Mell et al. 2015, Bercik et al. 2011, Bruce-Keller et al. 2015), but limited data from rats raised in non-sterile environments are available. Thus, several other experimental parameters could be explored by future studies, such as amount and preparation of feces or frequency and duration of treatments, which may improve the transmissibility of the strain-characteristic bacteria. Another limitation of the present study is the fact that FMT was not performed in anaerobic conditions, which may cause the loss of anaerobic strains. Moreover, our results cannot be generalized to FSL/FRL rats of a different age or gender, as these factors may change the microbial composition (Rodriguez et al. 2015, Haro et al. 2016). Microbial abundances also vary considerably between species, and the sparsity of studies within the gut-brain axis in rats with intact microbiota further complicates comparisons to previous work. However, rats raised in non-sterile environments may provide a more translational approach compared with, e.g., germ-free mice. Furthermore, rats were proposed to be more representative of the human gut microbiota than mice (Nguyen et al. 2015, Wos-Oxley et al. 2012), highlighting the importance of investigating the effects of FMT in rats rather than mice. Despite greater similarities between rat and human microbiota, the diversity of the rat intestinal microbiome was found to be 2–3 times higher than in humans, which may be due to a higher complexity in the rat gut bacterial ecosystem to harvest more nutrient from the basic laboratory diet (Manichanh et al. 2010). Thus, it is unknown if our results are comparable to a clinical population. We chose not to administer antibiotics prior to transplantation, as previous studies have shown that the establishment of donor phylotypes is not increased by prior antibiotic depletion (Manichanh et al. 2010). However, other studies have found behavioral and metabolic results following antibiotic treatment (Kelly et al. 2016), and we cannot exclude that microbiota depletion may have exerted differential effects in the present study. It was assumed that by performing repetitive gavage for several days, the gut microbiome community and structure would have reached its final equilibrium. As no longitudinal data were obtained during the FMT, we cannot conclude whether not observing differences in the gut microbiome of the different treatments was due to not starting with a low abundance microbiome, not performing the FMT long enough, or because there were indeed no differences. To reduce the number of animals used in this study, we used the same animals as donors and recipients, and, as a consequence, we transplanted fecal pellets. While the cecum may resemble the gut microbiota more closely, transplanting and analyzing feces may hold more translational value to humans. Finally, only recipient feces were analyzed in the present study, but the additional analysis of donor feces may provide additional mechanistic insights in future studies.

### Conclusion

This is the first study characterizing the gut microbiota community in FSL and FRL rats and transplanting a potentially strain-specific microbial profile from one rat strain to another. We found several rat strain differences in their bacterial profile, and some of these differences were comparable to those found in depressed patients (e.g., the increase in *Proteobacteria* and *Blautia*), while others are novel findings. The fecal transplant did not shift the profile towards the transplanted strain (i.e., FSL rats receiving FRL feces still had a more similar behavioral and microbial profile to FSL and not FRL rats). Fecal administration independent of the donor strain seemed to generally decrease mobility in the forced swim test compared to saline, whereas mobility in the OF was not affected. Future studies should investigate other animal models of depression and the consequences of FMT on behavior and microbiota. Moreover, the functional role of strain-characteristic microbiota should be elucidated in more detail.

## Electronic supplementary material

Supplementary Figure 1 Rarefaction curve displaying the number of observed OTUs as a function of sequencing depth. Note the large differences in scale between the x and *y*-axis. (PNG 53 kb)

High resolution image (TIF 75 kb)

Supplementary Figure 2 PCA plot showing the separation between FRL and FSL rats along the second component. Component 1: R^2^(x) = 0.251; component 2: R^2^(X) = 0.087. (PNG 1407 kb)

High resolution image (TIF 2774 kb)

Supplementary Table 1 The file contains the different OTUs that were identified in all samples, their abundances and taxonomic assignment. Each OTU is identified by a name (e.g. OTU_1) and the corresponding DNA sequence of the specific OTU can be found in the file. (XLSX 714 kb)

Supplementary Table 2 Overview of the significantly different bacterial species in FSL vs. FRL rats at OTU level. (XLSX 17 kb)

## Abbreviations

FMT: Fecal microbiota transplantation
FRL: Flinders resistant line
FSL: Flinders sensitive line

## References

[CR1] Abautret-Daly Áine, Dempsey Elaine, Parra-Blanco Adolfo, Medina Carlos, Harkin Andrew (2017). Gut–brain actions underlying comorbid anxiety and depression associated with inflammatory bowel disease. Acta Neuropsychiatrica.

[CR2] Abildgaard A, Elfving B, Hokland M, Lund S, Wegener G (2017). Probiotic treatment protects against the pro-depressant-like effect of high-fat diet in Flinders sensitive line rats. Brain Behav Immun.

[CR3] Aizawa E, Tsuji H, Asahara T, Takahashi T, Teraishi T, Yoshida S, Ota M, Koga N, Hattori K, Kunugi H (2016). Possible association of Bifidobacterium and Lactobacillus in the gut microbiota of patients with major depressive disorder. J Affect Disord.

[CR4] Akkasheh, G., Kashani-Poor, Z., Tajabadi-Ebrahimi, M., Jafari, P., Akbari, H., Taghizadeh, M., Memarzadeh, M. R., Asemi, Z. & Esmaillzadeh, A. 2015. Clinical and metabolic response to probiotic administration in patients with major depressive disorder: a randomized, double-blind, placebo-controlled trial. Nutrition 10.1016/j.nut.2015.09.003 26706022

[CR5] Albertsen M, Karst SM, Ziegler AS, Kirkegaard RH, Nielsen PH (2015). Back to basics--the influence of DNA extraction and primer choice on phylogenetic analysis of activated sludge communities. PLoS One.

[CR6] Bailey MT, Dowd SE, Galley JD, Hufnagle AR, Allen RG, Lyte M (2011). Exposure to a social stressor alters the structure of the intestinal microbiota: implications for stressor-induced immunomodulation. Brain Behav Immun.

[CR7] Bajaj JS, Hylemon PB, Ridlon JM, Heuman DM, Daita K, White MB, Monteith P, Noble NA, Sikaroodi M, Gillevet PM (2012). Colonic mucosal microbiome differs from stool microbiome in cirrhosis and hepatic encephalopathy and is linked to cognition and inflammation. Am J Physiol Gastrointest Liver Physiol.

[CR8] Bangsgaard Bendtsen KM, Krych L, Sorensen DB, Pang W, Nielsen DS, Josefsen K, Hansen LH, Sorensen SJ, Hansen AK (2012). Gut microbiota composition is correlated to grid floor induced stress and behavior in the BALB/c mouse. PLoS One.

[CR9] Benjamini Y, Hochberg Y (1995). Controlling the false discovery rate - a practical and powerful approach to multiple testing. J R Stat Soc.

[CR10] Bercik P, Denou E, Collins J, Jackson W, Lu J, Jury J, Deng Y, Blennerhassett P, Macri J, Mccoy KD, Verdu EF, Collins SM (2011). The intestinal microbiota affect central levels of brain-derived neurotropic factor and behavior in mice. Gastroenterology.

[CR11] Bharwani A, Mian MF, Foster JA, Surette MG, Bienenstock J, Forsythe P (2016). Structural & functional consequences of chronic psychosocial stress on the microbiome & host. Psychoneuroendocrinology.

[CR12] Bojanova DP, Bordenstein SR (2016). Fecal transplants: what is being transferred?. PLoS Biol.

[CR13] Bolger AM, Lohse M, Usadel B (2014). Trimmomatic: a flexible trimmer for Illumina sequence data. Bioinformatics.

[CR14] Bruce-Keller AJ, Salbaum JM, Luo M, Blanchard ET, Taylor CM, Welsh DA, Berthoud HR (2015). Obese-type gut microbiota induce neurobehavioral changes in the absence of obesity. Biol Psychiatry.

[CR15] Cammarota G, Ianiro G, Tilg H, Rajilic-Stojanovic M, Kump P, Satokari R, Sokol H, Arkkila P, Pintus C, Hart A, Segal J, Aloi M, Masucci L, Molinaro A, Scaldaferri F, Gasbarrini G, Lopez-Sanroman A, Link A, De Groot P, De Vos WM, Hogenauer C, Malfertheiner P, Mattila E, Milosavljevic T, Nieuwdorp M, Sanguinetti M, Simren M, Gasbarrini A (2017). European consensus conference on faecal microbiota transplantation in clinical practice. Gut.

[CR16] Canani RB, Costanzo MD, Leone L, Pedata M, Meli R, Calignano A (2011). Potential beneficial effects of butyrate in intestinal and extraintestinal diseases. World J Gastroenterol.

[CR17] Caporaso JG, Lauber CL, Walters WA, Berg-Lyons D, Lozupone CA, Turnbaugh PJ, Fierer N, Knight R (2011). Global patterns of 16S rRNA diversity at a depth of millions of sequences per sample. Proc Natl Acad Sci U S A.

[CR18] Christian LM, Galley JD, Hade EM, Schoppe-Sullivan S, Kamp Dush C, Bailey MT (2015). Gut microbiome composition is associated with temperament during early childhood. Brain Behav Immun.

[CR19] Dinan TG, Stanton C, Cryan JF (2013). Psychobiotics: a novel class of psychotropic. Biol Psychiatry.

[CR20] Edgar RC (2013). UPARSE: highly accurate OTU sequences from microbial amplicon reads. Nat Methods.

[CR21] Fung TC, Olson CA, Hsiao EY (2017). Interactions between the microbiota, immune and nervous systems in health and disease. Nat Neurosci.

[CR22] Galley JD, Yu Z, Kumar P, Dowd SE, Lyte M, Bailey MT (2014). The structures of the colonic mucosa-associated and luminal microbial communities are distinct and differentially affected by a prolonged murine stressor. Gut Microbes.

[CR23] Haro C, Rangel-Zuniga OA, Alcala-Diaz JF, Gomez-Delgado F, Perez-Martinez P, Delgado-Lista J, Quintana-Navarro GM, Landa BB, Navas-Cortes JA, Tena-Sempere M, Clemente JC, Lopez-Miranda J, Perez-Jimenez F, Camargo A (2016). Intestinal microbiota is influenced by gender and body mass index. PLoS One.

[CR24] Huang Ruixue, Wang Ke, Hu Jianan (2016). Effect of Probiotics on Depression: A Systematic Review and Meta-Analysis of Randomized Controlled Trials. Nutrients.

[CR25] Jiang H, Ling Z, Zhang Y, Mao H, Ma Z, Yin Y, Wang W, Tang W, Tan Z, Shi J, Li L, Ruan B (2015). Altered fecal microbiota composition in patients with major depressive disorder. Brain Behav Immun.

[CR26] Kelly JR, Borre Y, O’Brien C, Patterson E, El Aidy S, Deane J, Kennedy PJ, Beers S, Scott K, Moloney G, Hoban AE, Scott L, Fitzgerald P, Ross P, Stanton C, Clarke G, Cryan JF, Dinan TG (2016). Transferring the blues: depression-associated gut microbiota induces neurobehavioural changes in the rat. J Psychiatr Res.

[CR27] Khan TJ, Ahmed YM, Zamzami MA, Mohamed SA, Khan I, Baothman OAS, Mehanna MG, Yasir M (2018). Effect of atorvastatin on the gut microbiota of high fat diet-induced hypercholesterolemic rats. Sci Rep.

[CR28] Li HL, Lu L, Wang XS, Qin LY, Wang P, Qiu SP, Wu H, Huang F, Zhang BB, Shi HL, Wu XJ (2017). Alteration of gut microbiota and inflammatory cytokine/chemokine profiles in 5-fluorouracil induced intestinal mucositis. Front Cell Infect Microbiol.

[CR29] Magoc T, Salzberg SL (2011). FLASH: fast length adjustment of short reads to improve genome assemblies. Bioinformatics.

[CR30] Manichanh C, Reeder J, Gibert P, Varela E, Llopis M, Antolin M, Guigo R, Knight R, Guarner F (2010). Reshaping the gut microbiome with bacterial transplantation and antibiotic intake. Genome Res.

[CR31] Marin IA, Goertz JE, Ren T, Rich SS, Onengut-Gumuscu S, Farber E, Wu M, Overall CC, Kipnis J, Gaultier A (2017). Microbiota alteration is associated with the development of stress-induced despair behavior. Sci Rep.

[CR32] Mcilroy, S. J., Saunders, A. M., Albertsen, M., Nierychlo, M., Mcilroy, B., Hansen, A. A., Karst, S. M., Nielsen, J. L. & Nielsen, P. H. 2015. MiDAS: the field guide to the microbes of activated sludge. *Database (Oxford),* 2015, bav062 10.1093/database/bav062 PMC4483311 26120139

[CR33] Mell B, Jala VR, Mathew AV, Byun J, Waghulde H, Zhang Y, Haribabu B, Vijay-Kumar M, Pennathur S, Joe B (2015). Evidence for a link between gut microbiota and hypertension in the Dahl rat. Physiol Genomics.

[CR34] Miller AH, Raison CL (2016). The role of inflammation in depression: from evolutionary imperative to modern treatment target. Nat Rev Immunol.

[CR35] Naseribafrouei A, Hestad K, Avershina E, Sekelja M, Linlokken A, Wilson R, Rudi K (2014). Correlation between the human fecal microbiota and depression. Neurogastroenterol Motil.

[CR36] Nguyen TL, Vieira-Silva S, Liston A, Raes J (2015). How informative is the mouse for human gut microbiota research?. Dis Model Mech.

[CR37] O’callaghan A, Van Sinderen D (2016). Bifidobacteria and their role as members of the human gut microbiota. Front Microbiol.

[CR38] O'mahony SM, Hyland NP, Dinan TG, Cryan JF (2011). Maternal separation as a model of brain-gut axis dysfunction. Psychopharmacology.

[CR39] Overstreet DH (1993). The Flinders sensitive line rats: a genetic animal model of depression. Neurosci Biobehav Rev.

[CR40] Overstreet DH, Wegener G (2013). The flinders sensitive line rat model of depression--25 years and still producing. Pharmacol Rev.

[CR41] Panasevich MR, Morris EM, Chintapalli SV, Wankhade UD, Shankar K, Britton SL, Koch LG, Thyfault JP, Rector RS (2016). Gut microbiota are linked to increased susceptibility to hepatic steatosis in low-aerobic-capacity rats fed an acute high-fat diet. Am J Physiol Gastrointest Liver Physiol.

[CR42] Park AJ, Collins J, Blennerhassett PA, Ghia JE, Verdu EF, Bercik P, Collins SM (2013). Altered colonic function and microbiota profile in a mouse model of chronic depression. Neurogastroenterol Motil.

[CR43] Rizzatti G, Lopetuso LR, Gibiino G, Binda C, Gasbarrini A (2017). Proteobacteria: a common factor in human diseases. Biomed Res Int.

[CR44] Rodriguez JM, Murphy K, Stanton C, Ross RP, Kober OI, Juge N, Avershina E, Rudi K, Narbad A, Jenmalm MC, Marchesi JR, Collado MC (2015). The composition of the gut microbiota throughout life, with an emphasis on early life. Microb Ecol Health Dis.

[CR45] Romijn AR, Rucklidge JJ, Kuijer RG, Frampton C (2017). A double-blind, randomized, placebo-controlled trial of Lactobacillus helveticus and Bifidobacterium longum for the symptoms of depression. Aust N Z J Psychiatry.

[CR46] Sekirov I, Russell SL, Antunes LC, Finlay BB (2010). Gut microbiota in health and disease. Physiol Rev.

[CR47] Slattery DA, Cryan JF (2012). Using the rat forced swim test to assess antidepressant-like activity in rodents. Nat Protoc.

[CR48] Tamanai-Shacoori Z, Smida I, Bousarghin L, Loreal O, Meuric V, Fong SB, Bonnaure-Mallet M, Jolivet-Gougeon A (2017). Roseburia spp.: a marker of health?. Future Microbiol.

[CR49] Tillmann S, Awwad HM, Eskelund AR, Treccani G, Geisel J, Wegener G, Obeid R (2018). Probiotics affect one-carbon metabolites and catecholamines in a genetic rat model of depression. Mol Nutr Food Res.

[CR50] Tillmann S, Pereira VS, Liebenberg N, Christensen AK, Wegener G (2017). ZL006, a small molecule inhibitor of PSD-95/nNOS interaction, does not induce antidepressant-like effects in two genetically predisposed rat models of depression and control animals. PLoS One.

[CR51] Wallace CJ, Milev R (2017). The effects of probiotics on depressive symptoms in humans: a systematic review. Ann General Psychiatry.

[CR52] Wang H, Lee IS, Braun C, Enck P (2016). Effect of probiotics on central nervous system functions in animals and humans - a systematic review. J Neurogastroenterol Motil.

[CR53] Wang Q, Garrity GM, Tiedje JM, Cole JR (2007). Naive Bayesian classifier for rapid assignment of rRNA sequences into the new bacterial taxonomy. Appl Environ Microbiol.

[CR54] Wong ML, Inserra A, Lewis MD, Mastronardi CA, Leong L, Choo J, Kentish S, Xie P, Morrison M, Wesselingh SL, Rogers GB, Licinio J (2016). Inflammasome signaling affects anxiety- and depressive-like behavior and gut microbiome composition. Mol Psychiatry.

[CR55] Wos-Oxley M, Bleich A, Oxley AP, Kahl S, Janus LM, Smoczek A, Nahrstedt H, Pils MC, Taudien S, Platzer M, Hedrich HJ, Medina E, Pieper DH (2012). Comparative evaluation of establishing a human gut microbial community within rodent models. Gut Microbes.

[CR56] Zheng P, Zeng B, Zhou C, Liu M, Fang Z, Xu X, Zeng L, Chen J, Fan S, Du X, Zhang X, Yang D, Yang Y, Meng H, Li W, Melgiri ND, Licinio J, Wei H, Xie P (2016). Gut microbiome remodeling induces depressive-like behaviors through a pathway mediated by the host's metabolism. Mol Psychiatry.

