# Supplementary figures and images for: Altered fecal microbiota composition in the Flinders sensitive line rat model of depression
Source: Psychopharmacology (Berl). 2018 Nov 23;236(5):1445–57. doi: 10.1007/s00213-018-5094-2 (PMC6599185; doi:10.1007/s00213-018-5094-2)

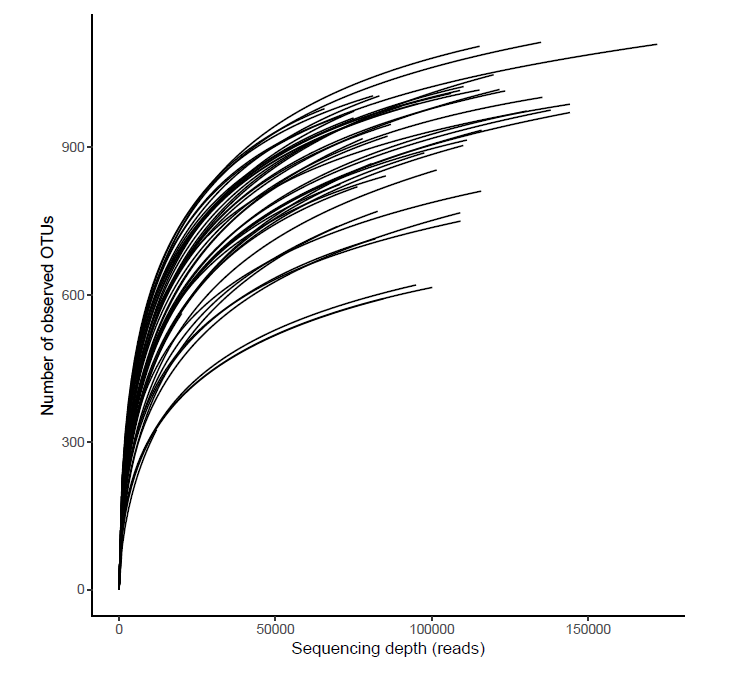

Supplement: Supplementary file 1 — Rarefaction curve displaying the number of observed OTUs as a function of sequencing depth. Note the large differences in scale between the x and y-axis. (PNG 53 kb) [file 213_2018_5094_Fig7_ESM.png]

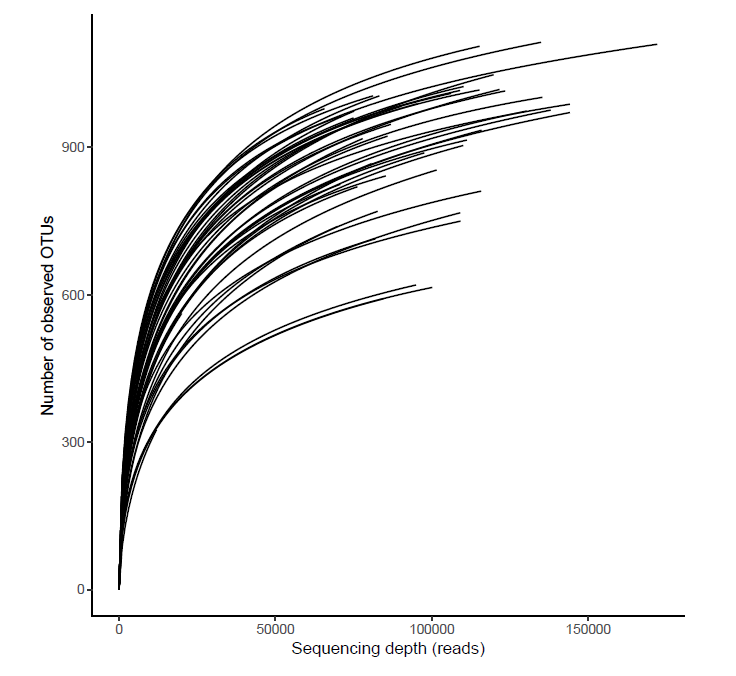

Supplement: Supplementary file 2 — High resolution image (TIF 75 kb) [file 213_2018_5094_MOESM1_ESM.tif]

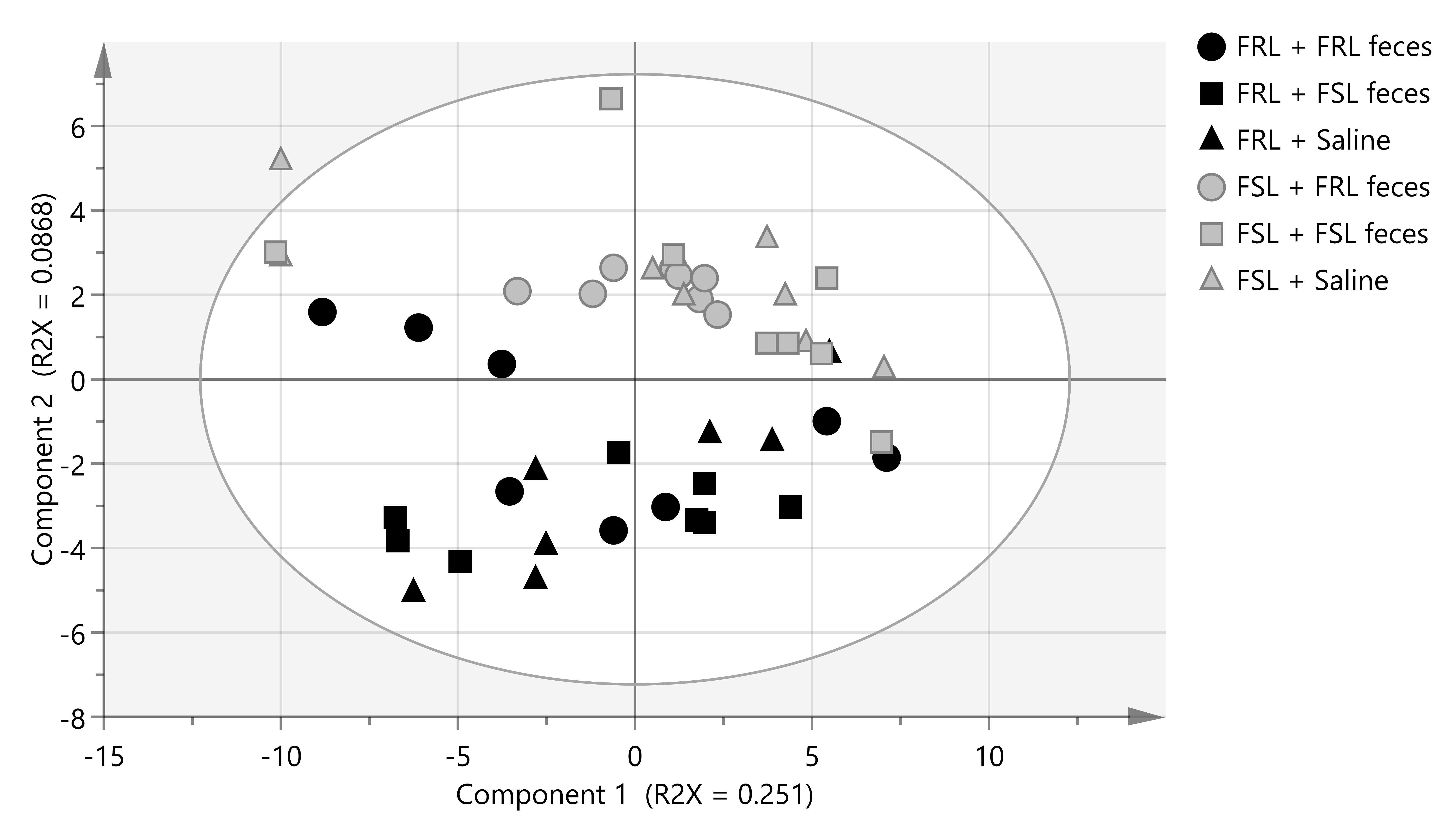

Supplement: Supplementary file 3 — PCA plot showing the separation between FRL and FSL rats along the second component. Component 1: R2(x) = 0.251; component 2: R2(X) = 0.087. (PNG 1407 kb) [file 213_2018_5094_Fig8_ESM.png]

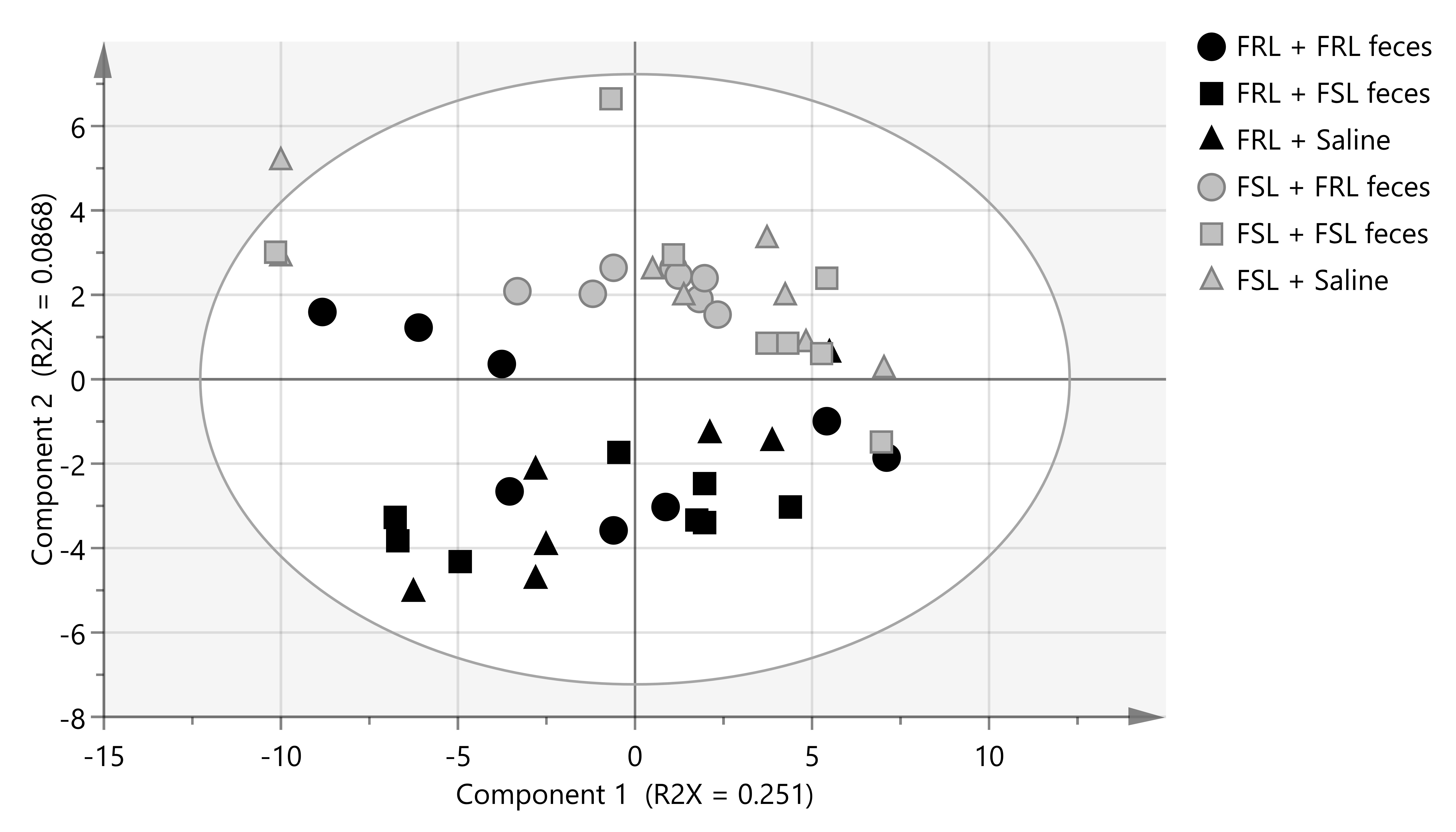

Supplement: Supplementary file 4 — High resolution image (TIF 2774 kb) [file 213_2018_5094_MOESM2_ESM.tif]
